# Affective temperament, job stress and professional burnout in nurses and civil servants

**DOI:** 10.1371/journal.pone.0176698

**Published:** 2017-06-06

**Authors:** Marcin Jaracz, Izabela Rosiak, Anna Bertrand-Bucińska, Maciej Jaskulski, Joanna Nieżurawska, Alina Borkowska

**Affiliations:** 1 Nicolaus Copernicus University in Torun, Faculty of Health Sciences, Chair of Clinical Neuropsychology, Bydgoszcz, Poland; 2 University of Warmia and Mazury. Department of Foreign Language Studies, Olsztyn, Poland; 3 Antoni Jurasz University Hospital No. 1, Bydgoszcz, Poland; 4 WSB University in Toruń, Management Department, Toruń, Poland; Technion Israel Institute of Technology, ISRAEL

## Abstract

**Introduction:**

The risk of professional burnout is constituted by job-related as well as individual factors. The latter involve affective temperament, which influences the perception of job-related stress. The aim of the present study was to assess the affective temperament, the level of job stress and professional burnout, as well as the relationships between these variables, in public servants and nurses.

**Material and methods:**

100 civil servants and 100 nurses were enrolled in the study. Affective temperament and burnout were assessed by means of TEMPS-A and MBI questionnaires, respectively. To measure the level of job-related stress, we have designed a 6-item self-reported questionnaire, which considered stressors common for both professions.

**Results:**

Compared to the civil servants, nurses showed higher rate of anxious temperament and experienced greater intensity of job-related stress. The groups did not differ in the intensity of burnout symptoms. The rates of cyclothymic and anxious temperaments correlated with the intensity of stress, and burnout symptoms in the group of nurses. Within the civil servants group, the level of stress correlated with intensity of burnout, however no correlations with affective temperament were observed. The regression analysis performed in both groups revealed the significant effect of stress and cyclothymic temperament on burnout, while the effect of anxious temperament was not significant.

**Conclusions:**

Cyclothymic and anxious temperaments are related to the level of experienced job stress and the risk of burnout. In professions like nursing, where employees show elevated rates of these temperaments, burnout prevention and stress management education is of particular importance.

## Introduction

Nurses and civil servants perform work which belongs to the public sector. Therefore, while carrying out their professional duties, the employees help citizens every day. In the case of nurses, it is taking care of patients by assisting them in activities of daily living, or serving them medicine. The civil servants’ work consists in helping citizens in cases where public administrative bodies can be of assistance, or in fulfilling their civic duties. Additionally, the common features of these professions include the necessity to keep high standards of contact with the patient or the applicant, and what follows, controlling ones behavior, performing ones duties in a hierarchical working environment, as well as having the potential risk of severe negative consequences taking place in the case of committing an error. Moreover, in Poland, the majority of workers of those professions receive similar salaries, which most of them consider insufficient [1]. The factor which differs in the two professions is the specificity of contact with the patient/applicant. The nurse encounters people who are hurting or dying, the contact is longer and more direct, which is a source of additional mental burden [2]. The features of the two aforementioned professions are source of chronic stress, which may cause some people to adapt to them in a unique manner, i.e. occupational burnout syndrome [3,4].

Occupational burnout is defined as a syndrome of exhaustion, depersonalization, and inefficacy, in reaction to chronic stress at workplace [5]. Despite the fact that currently it is considered that the phenomenon may affect workers of all professions, both traditional approach to the syndrome, as well as large number of studies suggest that people whose jobs involve helping others are especially susceptible [6,7,8]. The risk of professional burnout is not equal for all workers in a given group, as it is also influenced by, apart from the factors connected with the type of job, individual factors such as temperament, and personality [9]. Unique, relatively stable personality or temperamental traits may condition the manner in which the person reacts to stress at work. Therefore, they may be treated as a predisposing factor, or a factor which protects against professional burnout [10]. For example, personality traits belonging to the Five Factor Model of Costa and McRae–emotional stability, extraversion, conscientiousness agreableness and openness decrease the risk of burnout [9]. Conversely, neuroticism defined as the tendency to experience distressing emotions such as fear and depression contributes to the occurrence of the burnout syndrome [11]. Personality and psychological factors may play stronger role in the occurrence of job-related psychopathology, than organizational and interpersonal factors [12].

Affective temperament is a set of five relatively stable, genetically conditioned styles of emotional reaction (temperaments), which may be the predisposing factor in occurrence of affective disorders (for their descriptions, see Table 1). In their mild, subaffective form, they may be adaptive in an evolutionary context, for example by subserving better adjustment to social and professional requirements [13,14]. For example, hyperthymic temperament facilitates leadership, over-involvement and robustness to stress, whereas depressive–devotion to work, which makes these traits desirable in professional settings. Subjects with anxious temperament are prone to worry about their own and others’ health and safety, which is suitable in nursing and other disciplines consisting in professionally helping others [14,15,16]. Affective temperaments may thus constitute a predisposition to perform certain jobs. They also affect the manner in which people respond to occupational stress [15,17]. Depressive, cyclothymic, irritable and anxious temperaments are related to poorer response to stress emerging from professional situations, whereas hyperthymic temperament promotes robustness to occupational stress [16]. Thus, they may be treated as factors affecting the risk of occupational burnout, however, no research has yet been done on the subject.

**Table 1:** Descriptions of five temperaments assessed by TEMPS-A.

Temperament	Features
**Depressive**	Pessimistic, skeptical, gloomy, incapable of fun, preoccupied with inadequacy, failure and negative events, given to worry, guilt-prone, conscientious and self-disciplining.
**Cyclothymic**	Social withdrawal alternating with uninhibited sociability, labile self-esteem, overconfidence alternating with low self- confidence, alternating periods of unusually high and low professional and creative productivity, unexplained tearfulness alternating with extreme joyfulness.
**Hyperthymic**	High activity, territoriality, leadership, risk-taking, stimulus-seeking stress-resistance, extroversion, grandiosity.
**Irritable**	Restless, dysphoric, broody, choleric.
**Anxious**	Worry about mundane matters, preoccupation with possible or present external dangers to oneself of one’s relatives, taking care of others.

Based on: Akiskal 1998; Akiskal, Akiskal 2005; Akiskal et al. 2005; Tei-Tominaga et al. 2012.

The aim of the present study was to evaluate the relationships between affective temperament, stress, and occupational burnout among nurses and civil servants. Considering the fact that nurse’s work requires empathy and sensitivity to suffering, we hypothesized that nurses would display higher rates of anxious temperament. We also expected that anxious temperament would be related to experiencing greater stress and occupational burnout. As a result, we expected that nurses would feel more stressed about their working conditions and they would display higher intensity of symptoms of occupational burnout.

## Subjects and methods

### Subjects

The first group enrolled in the present study consisted of 100 civil service workers (36 male, 64 female), aged 22–67 years (mean 40 ±11), of which 87 subjects received higher education and the other 13—secondary education. The mean seniority in this group was 17.03±9.87 years of service. The second group included 100 nurses (27 male, 73 female), aged 22–62 years (mean 40±13). 65 of them received higher education, the other 35 received secondary education, and their mean seniority was 18.52±8.61 years. The civil servants group was recruited from among the staff of Kuyavian-Pomeranian Voivodeship Office in Bydgoszcz, Poland. These were mainly the workers of Citizens’ Affairs Department and Crisis Management Department. Citizens’ Affairs Department workers provide help for the citizens in obtaining their personal identification documents, or in affairs concerning Polish citizenship. Subjects working in Crisis Management Department receive notifications about emergency situations on the area of the voivodeship, and co-operate with the Voivodeship Emergency Unit in serving emergency medical help. Nurses were recruited from different wards of Antoni Jurasz University Hospital No. 1 in Bydgoszcz, Poland. We included only nurses whose job concerned direct contact with the patient. We did not consider nurses working in ward or hospital administration units. Information about the aim and study procedure was distributed in the aforementioned units. Subjects who expressed their willingness to participate in the study were approached by the research team member and handed envelopes including study questionnaires. All the subjects gave their written consent to participate in the study after the nature of the procedures had been explained to them. The study received approval from the Nicolaus Copernicus University in Torun, Collegium Medicum Bioethics Committee.

### Methods

#### TEMPS-A

Affective temperament was assessed by means of the Temperament Evaluation of Pisa, Paris and San Diego-autoquestionnaire version (TEMPS-A). TEMPS-A is a 110-item yes-or-no self-report autoquestionnaire, designed to assess affective temperament in psychiatric and healthy subjects. It consists of five sub-scales: depressive, cyclothymic, irritable, hyperthymic and anxious [18]. In the present study, we used the Polish version of TEMPS-A, validated in the sample of 521 Polish undergraduate students [19].

#### Job-related stress questionnaire

To evaluate participants’ level of job related stress, we have designed the 6-item self-reported questionnaire, which included one general question: „How do you rate your job stress in relationship to the following factors?” The participants were asked to answer that question in relationship to the following stressors: conflicts in the workplace, unpredictable events during worktime, salaries, the necessity to make important decisions, responsibility for others and fear of losing the job. The subjects rated their responses on 0–5 scale, with “0” indicating no stress and “5” indicating maximal level of stress.

#### MBI

The level of professional burnout was assessed by means of the Maslach Burnout Inventory [5]. MBI is a 22 item autoquestionnaire, addressing three general scales: emotional exhaustion (EE, 9 items), depersonalization (D, 5 items) and personal accomplishment (PA, 8 items). Subjects rate their job-related feelings/attitudes on 7 point scale according to the frequency of their occurrence, from 0 indicating “never” to, 7 indicating “every day”. In the present study, we analyzed the overall score as well as the score, obtained on each of the three scales. We utilized the Polish version of the scale, in adaptation by Pasikowski [20].

### Statistical analysis

Statistical analyses were performed with Statistica 10.0 program. Normality of distribution of analyzed variables was assessed with Shapiro-Wilk test. As the normality criterion had not been met, non-parametric tests were used during the analyses. Between-group differences were assessed with Mann-Whitney U test and correlations between variables were assessed by means of Spearman’s rho coefficient.

## Results

Sociodemographic data from both groups are shown in Table 2. No between-group differences were found with respect to age, sex distribution and seniority. However, in the civil servants group, the number of subjects holding higher degree of education was higher than in nurses.

**Table 2:** Sociodemographic data.

	Civil servants	Nurses	Statistic
**Age**	40 ±11	40±13	Student t: *p<*0.26
**Sex (% male)**	36	27	χ^2^: p<0.18
**Education (% higher education)**	87	65	χ^2^: *p*<0.01
Seniority (years)*	17.03±9.87	18.52±8.61	Student t: *p<*0.38

*- years working in public service/healthcare

The assessment of affective temperament in both groups (Table 3.) revealed higher rates of anxious temperament in nurses, when compared to civil servants (*U* = 864.00; *p*<0.04). We also analyzed between-sex differences in TEMPS-A scores within both groups. Males from the civil servants group scored lower on anxious scale compared to females (p<0.04). Female nurses scored lower on hyperthymic scale, than did male nurses (p<0.04).

**Table 3:** Affective temperament in civil servants and nurses.

	Civil servants		Nurses		P*
	Mean±SD	Median (25–75%)	Mean±SD	Median (25–75%)	
Depressive	0.26±0.12	0.23 (0.19÷0.28)	0.28±0.11	0.29 (0.19÷0.33)	0.57
Cyclothymic	0.30±0.22	0.28 (0.14÷0.42)	0.28±0.21	0.21 (0.09÷0.42)	0.42
Hyperthymic	0.47±0.21	0.45 (0.33÷0.61)	0.47±0.17	0.47 (0.33÷0.60)	0.75
Irritable	0.21±0.23	0.14 (0.04÷0.28)	0.18±0.18	0.09 (0.05÷0.24)	0.42
Anxious	0.26±0.22	0.19 (0.07÷0.38)	0.32±0.22	0.27 (0.13÷0.48)	0.03

*- Mann-Whitney U

We next sought to assess the intensity of self-reported stress indicated by the results on the job-related stress questionnaire (Table 4.). Nurses reported higher stress on all six items of the questionnaire, which also translated to higher overall score in the questionnaire. Furthermore, female nurses reported higher stress due to interpersonal conflicts, than male nurses (p<0.01). They also received higher overall score in the questionnaire (p<0.03). No between-sex differences were found in the civil servants group.

**Table 4:** Professional burnout in civil servants and nurses.

	Civil servants (n = 100)		Nurses (n = 100)		
	Mean±SD	Median (25–75%)	Mean±SD	Median (25–75%)	P*
**Emotional exhaustion**	1.07±0.61	1.11 (0.56÷0.14)	0.98±0.57	1.00 (0.56÷1.33)	0.33
**Depersonalization**	0.82±0.61	0.80 (0.20÷1.20)	0.73±0.54	0.80 (0.20÷1.00)	0.34
**Self accomplishment**	1.84±0.47	1.87 (1.62÷2.12)	1.81±0.44	1.81 (1.56÷2.00)	0.65
**General score**	1.24±0.36	1.18 (0.99÷1.44)	1.17±0.26	1.16 (0.96÷1.33)	0.26

*- Mann-Whitney U

We found no differences between civil servants and nurses in the intensity of burnout symptoms as evidenced by the scores on MBI scale (Table 5.). Female nurses reported lower self-accomplishment, than male nurses (p<0.02). We found no differences within the civil servants group.

**Table 5:** Job-related stress in civil servants and nurses.

	Civil servants (n = 100)		Nurses (n = 100)		
	Mean±SD	Median (25–75%)	Mean±SD	Median (25–75%)	p*
**General score**	2.72±1.06	2.83 (2.00÷3.66)	3.87±0.54	3.83 (3.58÷4.25)	0.001
**Workplace conflicts**	2.25±1.37	2.00 (1.00÷3.00)	3.39±1.14	3.00 (2.50÷4.00)	0.001
**Unpredictable events**	2.73±1.40	3.00 (2.00÷4.00)	3.86±1.01	4.00 (3.00÷5.00)	0.001
**Salaries**	3.12±1.62	3.00 (2.00÷5.00)	4.16±0.97	4.00 (3.00÷5.00)	0.001
**Important decisions**	2.78±1.54	3.00 (1.50÷4.00)	3.90±1.05	4.00 (3.00÷5.00)	0.001
**Responsibility for others**	2.65±1.65	3.00 (1.00÷4.00)	3.75±1.09	4.00 (3.00÷5.00)	0.001
**Fear of losing the job**	2.82±1.61	3.00 (2.00÷4.00)	4.18±1.00	5.00 (4.00÷5.00)	0.001

*- Mann-Whitney U

The correlational analyses revealed relationships between the traits of affective temperament, stress and burnout. In the group of nurses, the rate of cyclothymic temperament was related to higher emotional exhaustion (rho = 0.29, *p*<0.01), depersonalization (rho = 0.25; *p*<0.01), general MBI score (rho = 0.29; *p*<0.01) and overall score in job-related stress questionnaire (0.22; *p*<0.02). The rate of anxious temperament was positively correlated with the intensity of emotional exhaustion (rho = 0.24; *p*<0.01) depersonalization (0.23; *p*<0.01), general MBI score (0.22; *p*<0.03) as well as with the general score in the job-related stress questionnaire (0.29; *p*<0.01). The global score on the job-related stress questionnaire was also found to be positively correlated with the general MBI score (rho = 0.25; *p*<0.02) as well as score on the emotional exhaustion sub-scale (rho = 0.32; *p*<0.01) in nurses, and depersonalization sub-scale in civil servants. In the civil servants group, no correlations between affective temperament and stress or burnout intensity, were found.

As both groups differed in the level of job-related stress and anxious temperament, both these variables being correlated with the level of burnout, their effect on the level of burnout, controlled for sociodemographic variables (age, sex, education and seniority), was assessed. To this end, a stepwise multivariate linear regression analysis was performed (Table 6). Age, sex, education, seniority, stress and anxious temperament were put into the model as predictors, and the level of burnout as dependent variable. The level of burnout was significantly predicted by the level of stress (*β* = 0.29; *p*<0.01) and education (*β* = 0.15; *p*<0.04). Effects of anxious temperament, sex, age and seniority were not significant.

**Table 6:** Results of stepwise multiple-regression of sociodemographic variables, affective temperament and stress on burnout.

Predictor variables	β	*t*	*p*
**Constant**	0.90	13.13	0.01
Age*	0.51	0.75	0.45
Sex*	0.10	1.53	0.12
Education*	0.15	2.09	0.03
Seniority*	0.01	0.07	0.94
Depressive*	-0.03	-0.45	0.64
**Cyclothymic**	0,24	2.54	0.01
Hyperthymic*	0.03	0.47	0.64
Irritable*	-0.01	-0.22	0.82
Anxious*	-0.03	1.39	0.75
**Overall** **stress**	0.07	3.89	0.01
**R**	0.29		
**R**^**2**^	0.08		
**Adjusted R**^**2**^	0.07		

*- variables excluded in the final step of the multiple regression analysis

## Discussion

The aim of the present study was to evaluate the relationships between affective temperament and work-related stress, and the intensity of professional burnout among nurses and civil servants. The results obtained confirm the relationship between the variables; however, this does not transfer to the differences in the intensity of burnout between the studied groups.

The results proved the hypothesis that the two professional groups show different profiles of affective temperament. As expected, the nurses displayed higher intensity of anxious temperament. While in its extreme form, the anxious temperament takes the form of anxiety disorder, in its lower, sub-clinical intensity, it manifests itself as the tendency to take care for one’s own and one’s families’ health and safety. Those features may be described as “altruistic anxiety”, which may be the predisposition to performing jobs aimed at helping others, especially, the suffering ones [21]. Despite the affective temperament no being researched in nurses so far, Our results seem consistent with research performed with the use of other tools evaluating temperament and personality. In the study by Sand [22] conducted with the use of tools for assessing personality, empathy and interpersonal sensitivity, it was shown that features of character connected with nursing are empathy, sensitivity to non-verbal communication, discomfort-proneness and service-mindedness. Additionally, Biagoli et al. [23] observed a high level of prosociality in nurses. Nurses displaying these features were highly valued by their supervisors. Thus, our research is yet another one that proved that a specific configuration of affective temperament traits may be connected with the predisposition to performing certain jobs [24]. In our previous study with emergency medicine professionals we have shown that the hyperthymic temperament, connected with resistance to stress and proneness to taking risks, is a desired feature in this profession. Higher intensity of hyperthymic temperament is connected with better performance of complicated tasks under time pressure, which is a situation often encountered by paramedics in their work [25].

In line with the hypotheses, nurses displayed higher intensity of occupational stress in all aspects evaluated by the Job-Related Stress Questionnaire. This happened, even though it included problems which are present in an equally intensive manner in the job of a nurse and a civil servant.

Higher intensity of experienced stress in nurses is partly related to the different specificity of the relation with the person being helped—nurses have a more direct relation, they help the suffering, sometimes the dying, which is a source of additional burden [2]. However, our correlational analyses indicate that these differences should also be looked at in the context of temperament. We have observed a relationship between the traits of affective temperament and the intensity of stress and occupational burnout. The correlations were only present in the nurses group, which may stem from the fact that the role of temperament and personality in job-related stress is bigger in nurses. The intensity of both stress and occupational burnout symptoms was related to the rates of cyclothymic and anxious temperament, which was higher in the nurses group. While the correlational analyses indicated the relationship between anxious and cyclothymic temperament and burnout, the regression analysis showed stress and cyclothymic temperament and to be the strongest predictors of burnout, while the effect of anxious temperament was not significant. A possible explanation of this discrepancy is that the effect of temperament on burnout may be indirect, with possible mediating role of stress. This hypothesis is supported by findings of Kikuchi et al. [26], who demonstrated that affective temperament influences depressive symptoms in nurses both directly and indirectly. In the latter case, this relationship is mediated by job stress. This indicates, that certain traits of affective temperament result in poorer coping with occupational stress, which increases the risk of depressive, or burnout symptoms.

Concordantly, cyclothymic and anxious temperaments were found to be connected with poorer coping. Sakai et al. [15] in a large group of Japanese company workers found the level of anxious temperament to be related to higher stress related to job demands and interpersonal conflicts, as well as poorer capacity to benefit from social support in the workplace. In a study by Nakai et al. [27], the level of anxious temperament predicted negative appraisal of stressful life events, which resulted in their negative effect on mood and well-being. Moreover, Tei-Tominaga et al. [17] demonstrated, that cyclothymic and anxious temperaments are related to higher risk of depressive symptoms occurring as a result of work-related stress. Thus, it may be assumed that higher intensity of both temperaments promotes a more negative assessment of work-related events, which makes them more stressful. This, in turn, increases the risk of occupational burnout.

We have also observed a positive correlation between the intensity of stress and occupational burnout. In both of the groups, the intensity of stress correlated with the general result of MBI. Numerous authors have proven the relationship between the intensity of stress and the risk of occupational burnout [28, 29]. Stress associated with the factors we included in the questionnaire—responsibility, interpersonal conflicts, and unpredictability was connected with increased risk of occupational burnout in medical and non-medical professions [30,31,32]. Chronic stress caused by the aforementioned factors may result in the feeling of discrepancy between the employee’s expectations and the actual situation at workplace, which in turn increases the risk of occupational burnout [33]. Increased stress correlated with increased depersonalization in the civil servants group, and with emotional exhaustion in the nurses group. Both of these phenomena have a negative impact on the quality of work provided by the helping professions. It has been observed that increased depersonalization and emotional exhaustion in nurses negatively correlates with the patients' satisfaction from the care they are given [34,35]. The phenomenon is also important for the work of civil servants–stress induced depersonalization may be reflected in lower quality of contact with the applicants, which will make doing their errands more difficult. It may also negatively influence the social perception of work of administrative offices. Finally, both depersonalization and emotional exhaustion negatively influence the mental health of workers. Pompili et al. [36] observed that these burnout dimensions increase the level of hopelessness, and thus, of suicide risk, in psychiatric nurses.

Despite the fact that nurses displayed higher intensity of occupational stress than civil servants and the intensity of stress was connected with more intense symptoms of occupational burnout, the groups did not differ in the results of the MBI questionnaire. This was probably due to the fact that the risk of occupational burnout in both groups is conditioned by slightly different factors. The civil servants work is more repetitive than that of a nurse, which is a source of stress that our questionnaire does not consider. This hypothesis is also confirmed by the fact that the correlation between the temperament and the intensity of occupational burnout was only observed in the nurses group.

Finally, in the civil servants group, subjects with higher education demonstrated lower intensity of occupational burnout, than those with secondary education. The phenomenon was observed despite the two groups differed in the intensity of experienced stress. This result is best explained by the hypothesis that subjects with higher education show more efficient coping; therefore, stress did not translate to symptoms of occupational burnout. That difference was not observed in nurses, which emphasizes the need for including education on stress coping and preventing occupational burnout in the curriculum of this profession.

The present study has several limitations. We enrolled nurses working on different wards, which makes this group heterogenous. It is important, as previous research indicate, that working on different wards is related to different risk of burnout. Furthermore, assessing subjects working in different wards or departments provides a possibility, that their work requires different amounts of time spent with their clients or patients. Provided that the professional helping relationship is a source of stress and, consequently, of burnout, it is important to control for this variable in further studies. Finally, cross-sectional design of the present study limits the possibility of drawing conclusions about causal links between temperamental variables, stress and burnout. Longitudinal study would enable to define risk-factors and to assess their influence on the development of burnout symptoms.

The results of the present study show two roles of anxious temperament in the context of professional work. It predisposes to jobs connected with helping others, but at the same time, as it is connected with the inclination to worry and higher interpersonal sensitivity, it may cause people to perceive work-related problems as more stressful. In human service professions, it may increase the risk of compassion fatigue, which more emphatic and self-giving people are prone to, and which is connected with the occurrence of occupational burnout [37,38].

Our study has also demonstrated a relationship between stress and symptoms of occupational burnout in both of the groups. Since the symptoms have negative influence on the quality of work of the helping professions, it is advised to monitor the stress level of the employees and to undertake actions aimed at limiting the sources of stress. Yet another possibility is to educate the staff on how to handle occupational stress—an action which has been shown to effectively counteract occupational stress.

## Supporting information

S1 Dataset (XLSX)
